# Terrestrial 3D laser scanning to track the increase in canopy height of both monocot and dicot crop species under field conditions

**DOI:** 10.1186/s13007-016-0109-7

**Published:** 2016-01-29

**Authors:** Michael Friedli, Norbert Kirchgessner, Christoph Grieder, Frank Liebisch, Michael Mannale, Achim Walter

**Affiliations:** Institute of Agricultural Sciences, ETH Zürich, Universitätstrasse 2, 8092 Zürich, Switzerland

**Keywords:** Laser scanning, Scan point cloud, Canopy height growth, Maize, Soybean, Wheat

## Abstract

**Background:**

Plant growth is a good indicator of crop performance and can be measured by different methods and on different spatial and temporal scales. In this study, we measured the canopy height growth of maize (*Zea mays*), soybean (*Glycine max*) and wheat (*Triticum aestivum*) under field conditions by terrestrial laser scanning (TLS). We tested the hypotheses whether such measurements are capable to elucidate (1) differences in architecture that exist between genotypes; (2) genotypic differences between canopy height growth during the season and (3) short-term growth fluctuations (within 24 h), which could e.g. indicate responses to rapidly fluctuating environmental conditions. The canopies were scanned with a commercially available 3D laser scanner and canopy height growth over time was analyzed with a novel and simple approach using spherical targets with fixed positions during the whole season. This way, a high precision of the measurement was obtained allowing for comparison of canopy parameters (e.g. canopy height growth) at subsequent time points.

**Results:**

Three filtering approaches for canopy height calculation from TLS were evaluated and the most suitable approach was used for the subsequent analyses. For wheat, high coefficients of determination (R^2^) of the linear regression between manually measured and TLS-derived canopy height were achieved. The temporal resolution that can be achieved with our approach depends on the scanned crop. For maize, a temporal resolution of several hours can be achieved, whereas soybean is ideally scanned only once per day, after leaves have reached their most horizontal orientation. Additionally, we could show for maize that plant architectural traits are potentially detectable with our method.

**Conclusions:**

The TLS approach presented here allows for measuring canopy height growth of different crops under field conditions with a high temporal resolution, depending on crop species. This method will enable advances in automated phenotyping for breeding and precision agriculture applications. In future studies, the TLS method can be readily applied to detect the effects of plant stresses such as drought, limited nutrient availability or compacted soil on different genotypes or on spatial variance in fields.

## Background

Plant growth is a good indicator of crop performance and is measureable by different methods and on different spatial and temporal scales [1]. Plant growth reveals detailed information about the state of a plant [2] and allows for the assessment of the tolerance of a plant to abiotic stress such as drought [3, 4], heat [4] or nutrient deficiency [5]. Today’s technologies offer many different possibilities to measure plant growth automatically, non-invasively and non-destructively. Many of these technologies construct a 3D scan point cloud of plants or canopies. A very simple approach to measure plant growth by taking images with a commercial digital camera was used for example by Sritarapipat et al. [6] to observe plant height changes in a rice field. In more complex approaches 3D images of plants are reconstructed by using stereo cameras [7], by analysing multiple images taken from different viewing angles [8] or by taking depth images [9]. In recent studies, unmanned aerial vehicles (UAVs) were used to generate 3D reconstructions of winter wheat from multiple images to estimate crop height [10] and to generate 3D digital surface models of barley from hyperspectral information [11]. In another study, a laser scanner was mounted on a UAV to estimate crop height of maize [12].

A very interesting and precise technology is the so-called 3D digitizer which uses ultrasonic or electromagnetic devices (digitizing pens) to construct 3D images of plant parts or whole plants. Plant architecture of different crops was measured with 3D digitizers to calculate light models in plant canopies in rice [13] and cucumber [14]. 3D digitizing is very labour and time intensive because the digitizing pen needs to be manually pointed to important landmarks on the plant (for example leaf and shoot tips) to map plant architecture in 3D. Therefore, this technology cannot be used as an automated, high throughput phenotyping system.

A sophisticated technology, that is becoming more and more important, is the active remote-sensing laser rangefinder which uses a laser beam to determine the distance to an object. Different principles for distance detection exist (for a review see [15] or [16]).

Terrestrial laser scanning (TLS) offers a unique opportunity to make non-invasive and non-destructive measurements of canopies to characterize plant growth and to analyze diverse architectural parameters. TLS measurements render point clouds that depict the surface of the visible canopy oriented towards the observing device. These point clouds can be further analyzed, which has been done already in the fields of (1) forest ecology; (2) precision agriculture; and (3) phenotyping.

So far, most TLS studies were conducted in forest ecology to measure tree height, volume, leaf area, biomass and other important plant parameters [17–19]. Hosoi and Omasa [20] for example investigated the seasonal change of broad-leaved woody canopy leaf area density profiles. The plant structure and chlorophyll content in broadleaf saplings was studied by Eitel et al. [21].

In precision agriculture, measurements of orchard volumes [22, 23] or leaf area in orchards [23] or viticulture [24] have been conducted. The geometric characterization of tree crops is important for a number of different aspects such as the application of pesticides or irrigation systems (see [15] for a review). The aspect of canopy characterization is also important in vineyards to improve pesticide application methods [25]. In field crops, TLS was applied to discriminate maize plants from weeds and soil for a targeted application of herbicides [26]. Sensing of the nitrogen status of wheat plants by TLS was used for improved application of nitrogen fertilizers [27]. In another approach, Saeys et al. [28] used TLS to estimate crop density of wheat that could be used to automatically adjust the speed of a combine harvester for a constant intake of biomass.

Another important research field in which TLS is applied is plant phenotyping under lab or field conditions. Morphological plant parameters such as canopy height [29, 30] and leaf area [31, 32] have been investigated. Besides morphological parameters also structural (number of leaves, orientation of surfaces, topology) and functional information (photosynthesis, stomatal conductance etc.) has been studied [33]. Biomass [29, 34, 35] is probably the second most important parameter next to height growth [30]. In a newer approach, the detection of individual maize plants has been performed to improve plant growth models or crop management strategies [36]. The relevance of TLS measurements for field research remained limited though, since TLS measurements were typically conducted on single plants in pots [37, 38] or on small plants like *Arabidopsis thaliana* [39] from which conclusions to crops cannot easily be drawn. Furthermore, these measurements were often carried out under controlled and relatively artificial environmental conditions such as in climate chambers [39] or greenhouses [32, 40]. If TLS measurements were conducted in the field, this was done on very small areas ([41] 1 m^2^) or with a low resolution [42].

**Fig. 1:**
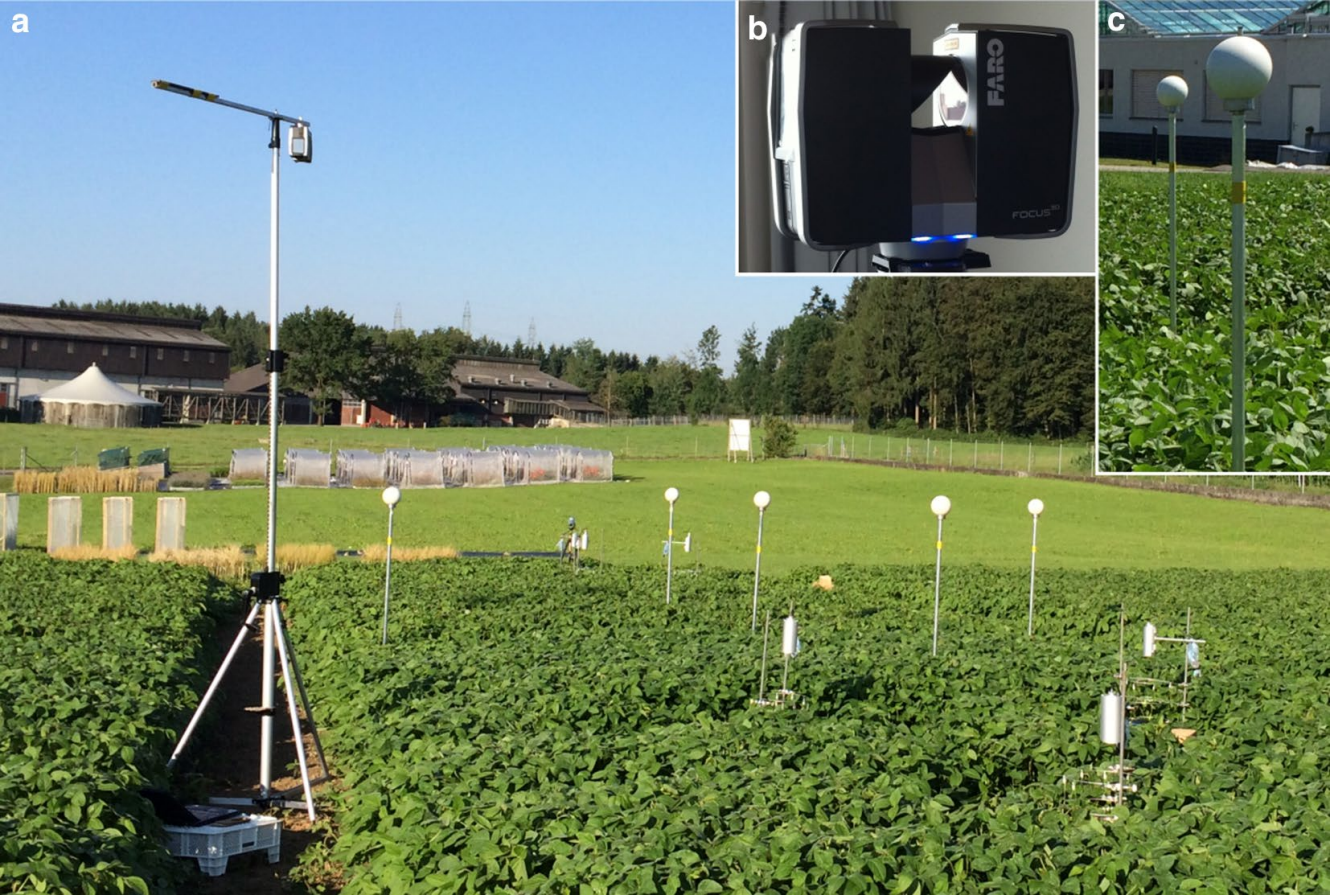
Experimental setup in the soybean field. **a** The setup in a soybean field with the laser scanner on an elevator tripod and *white* spherical targets to merge the single scans into a 3D point cloud; **b** close-up view of “Faro Focus 3D S 120” laser scanner; **c** close-up view of *white* spherical targets on aluminium rods

**Fig. 2:**
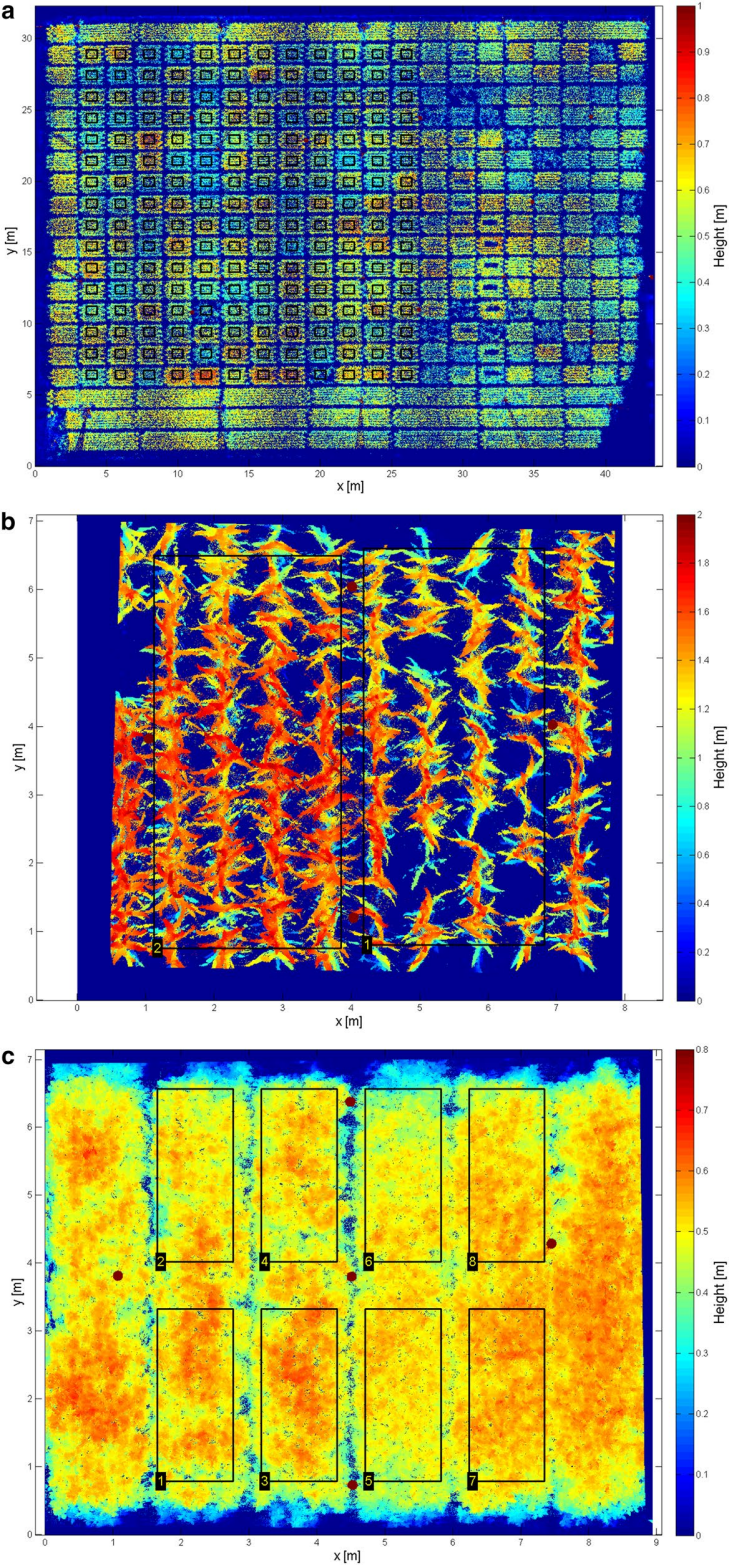
Plant height maps (*bird’s eye view*) computed from TLS. *Black* *rectangles* indicate ROIs and *red circles* the spherical targets. **a** Wheat field; **b** maize field; **c** soybean field

Elucidation of improved field management practices or of optimal genotypes in breeding programs needs to be done in plots and plant canopies of a relevant size in the field. Therefore, it was the overall aim of this study to analyze the capability and the limits of TLS approaches (Fig. 1) in the field on plot areas of several dozen to hundreds of m^2^ in different crops (Fig. 2; wheat, maize and soybean) that are of relevance to global agriculture. Precise knowledge of these capabilities and limits is necessary to better connect the multitude of small-scale experiments under controlled conditions with field studies and to come to conclusions of relevance for crop science with respect to the grand challenges of global climate change and sustainable intensification of agricultural practices.

Therefore, we tested the hypotheses, whether TLS field measurements are capable to elucidate (1) differences in architecture that exist between genotypes; (2) genotypic differences in canopy height growth during the season and (3) short-term growth fluctuations (within 24 h), which could indicate e.g. responses to rapidly fluctuating environmental conditions.

## Results

### Correlation between manually and TLS-derived wheat canopy height

**Fig. 3:**
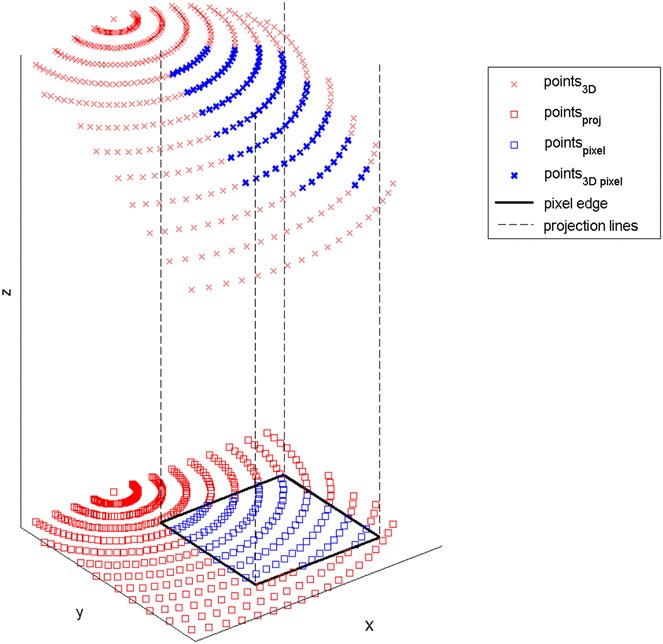
Calculation principle of height maps and statistics. In a first step, 3D points (points_3D) are projected to *xy-plane* (points_proj). Then, for each ROI (pixels for height maps) the contained points_proj are determined. Then, the *z-coordinates* of points in 3D which correspond to the ROI are known and can finally be used for further processing or statistical evaluation

**Fig. 4:**
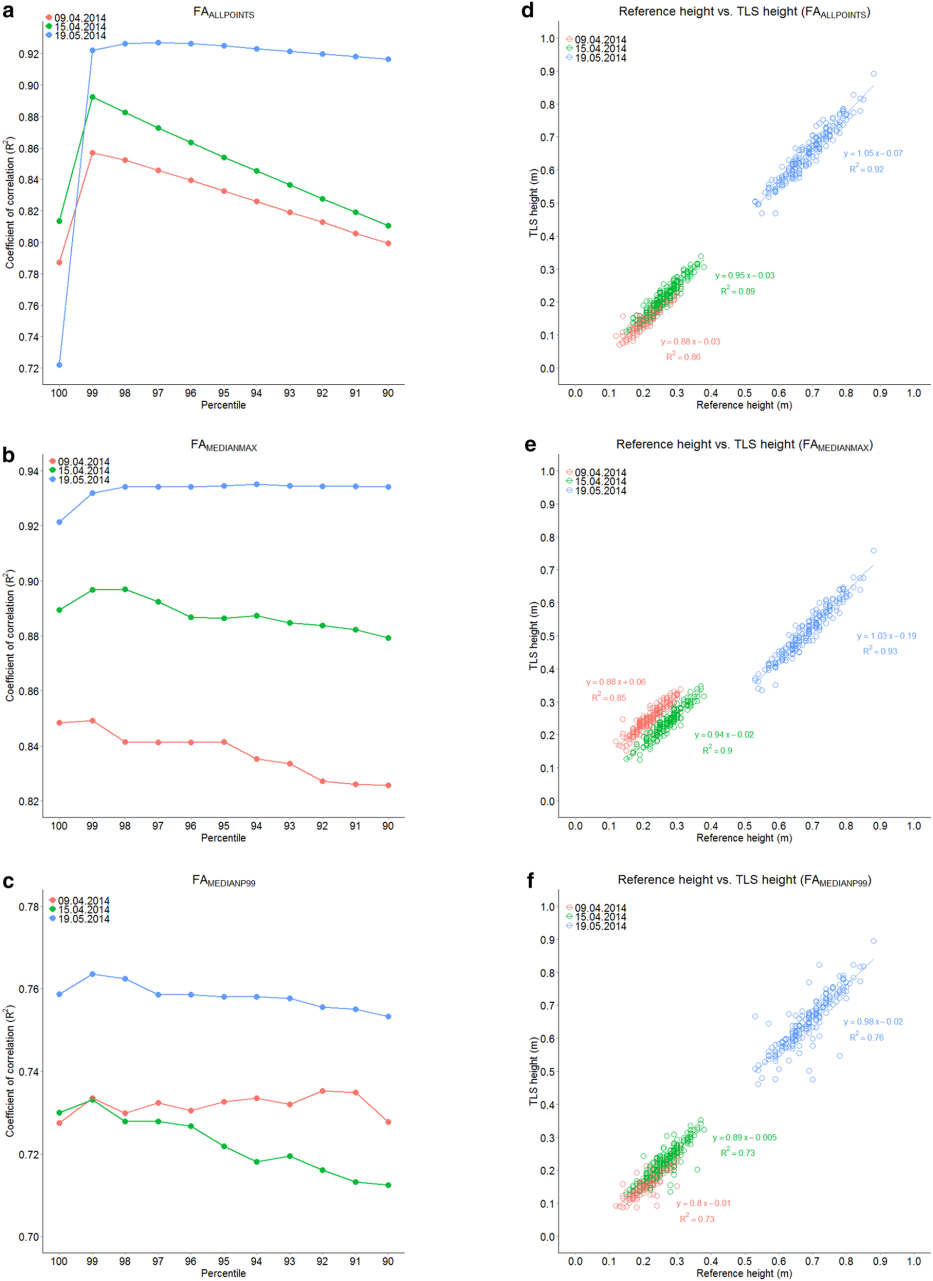
Correlation between manually measured and TLS-derived canopy height of *T. aestivum* for three dates. Coefficients of correlation for manually measured and TLS-derived canopy height of *T. aestivum* shown for the 100th to 90th percentile for three filtering approaches (FA): **a** FA_ALLPOINTS_; **b** FA_MEDIANMAX_; **c** FA_MEDIANP99_. Regression between manually measured (reference height) and TLS-derived canopy height (calculated from the 99th percentile for each FA) of *T. aestivum*: **d** FA_ALLPOINTS_; **e** FA_MEDIANMAX_; **f** FA_MEDIANP99_, n = 192 per date

Conceivably, outliers at the top of the raw data point cloud can lead to erroneous interpretations of canopy height (Fig. 3). Therefore, three filtering approaches were conducted with the aim to identify optimal filtering approaches for subsequent tasks (see “Methods” for more details). In order to perform this quality check, the coefficients of determination (R^2^) of the linear correlations between the manually measured reference height and three filtering approaches (FAs) for the TLS-derived canopy height were evaluated for the 100th (P100) to the 90th percentile (P90) of the investigated regions of interest (ROIs) for three measurement dates (Fig. 4a–c). For the three FAs, R^2^ reached highest values for the last measurement date. At this date, the canopies of the wheat plots were denser and reached canopy closure. Therefore, the laser beam could not penetrate very deep into the canopies. Thus, most of the scan points were located on top of the canopy.

The investigated ROIs were analyzed by testing three filtering approaches with different percentiles. The first FA used all contained scan points of each ROI and calculated certain percentiles of the z coordinate. The second FA filtered the ROI patchwise by a weighted median of certain percentiles. Thereby, the full ROI was considered, even if extreme values were present as for example in very heterogeneous field situations with a few very high plants. See “Data processing and data analysis” Sect. for a detailed description of the calculation of FAs. In the FA, which included all points of a ROI in the calculation (FA_ALLPOINTS_), R^2^ for P100 was lowest for all measurement dates (Fig. 4a). This percentile included all maximum points and as a consequence also potential outliers that contributed to the low R^2^. The values of R^2^ for the first two measurement dates gradually decreased from P99 to P90. At these two dates, the canopy was not yet closed, so that the laser beam reached lower parts of the canopy. Thus, the values of the lower percentiles did not depict the maximum but lower parts of the canopy.

In the FA using the weighted median of the maxima of each patch in a ROI (FA_MEDIANMAX_), R^2^ values of P100 for the three dates were also slightly lower than the ones of P99. R^2^ values for the last measurement date did not markedly vary between P99 and P90. The values of R^2^ for the first two measurement dates decreased from P99 to P90 and were always lower for the first date.

In the FA using the weighted median of P99 of each patch in a ROI (FA_MEDIANP99_) the highest values for R^2^ were obtained for the last measurement. However, the values were clearly lower for this FA compared to the other two FAs. The values for R^2^ of the first two dates were very similar from P100 to P96 but then diverged with decreasing percentiles below P95. The values of R^2^ for the first measurement were more or less uniform throughout the entire tested range of R^2^, whereas the values of R^2^ for the second measurement date decreased towards lower percentiles.

For the three evaluated FAs, P99 resulted in the highest values for R^2^ throughout the season. Therefore, P99 was considered as the TLS measure which approximated canopy height most realistically. In the next step, FAs were compared with each other on the basis of P99 values of TLS-derived canopy height that were plotted against manually measured reference heights (Fig. 4d–f). For FA_ALLPOINTS_, R^2^ increased from 0.86 for the first date to 0.92 for the last date (Fig. 4d). For FA_MEDIANMAX_, R^2^ increased from 0.85 for the first date to 0.93 for the last date (Fig. 4e). For FA_MEDIANP99_, R^2^ was comparable for the first (R^2^ = 0.73) and the second date (R^2^ = 0.73) but was higher (R^2^ = 0.76) for the third date (Fig. 4f). Using the three dates combined in a regression, FA_ALLPOINTS_ (R^2^ = 0.99) and FA_MEDIANP99_ (R^2^ = 0.98) reflect higher coefficients of determination than FA_MEDIANMAX_ (R^2^ = 0.95). Therefore, FA_ALLPOINTS_ was used in subsequent calculations.

**Fig. 5:**
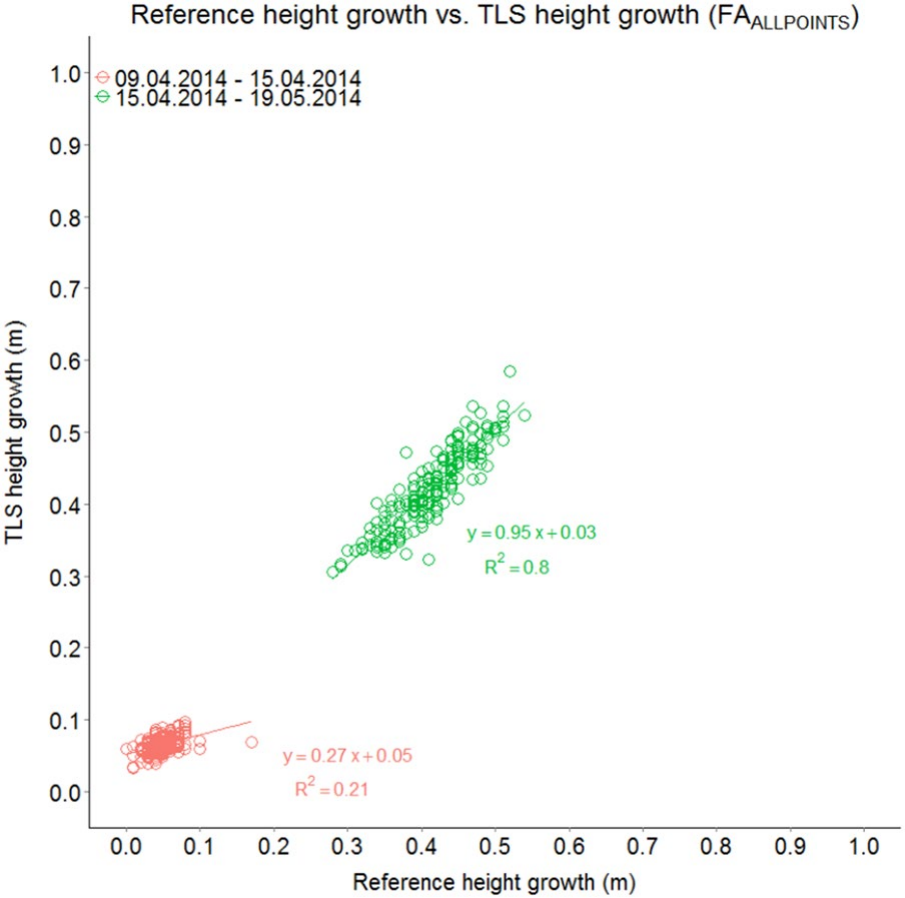
Correlation between canopy height growth of *T. aestivum* from manually measured (reference) and TLS-derived canopy height. Canopy height growth was calculated for the periods from 09 Apr 2014 to 15 Apr 2014 and 15 Apr 2014 to 19 May 2014 using FA_ALLPOINTS_, n = 192 per date

Using FA_ALLPOINTS_, the correlation between manually and TLS-derived canopy height growth was then analyzed for wheat as well. Growth can be calculated by the difference of the canopy height for a certain plot at two subsequent dates. Differences of manual height measurements and differences of TLS-derived canopy height were then put in relation to each other for different measurement periods. For the period from the first to the second measurement, the increase in canopy height was not large, which resulted in a relatively low R^2^ of 0.21 (Fig. 5). For the period from the second to the third measurement, the increase in canopy height for all genotypes was—according to the manual reference measurements—between 0.2 and 0.6 m. For this period, a high R^2^ of 0.80 was obtained for FA_ALLPOINTS_ (Fig. 5). It has to be noted that the values for the first period seem to be approximated by the correlation obtained for the second period in a reliable manner.

### Short-term canopy height growth

**Fig. 6:**
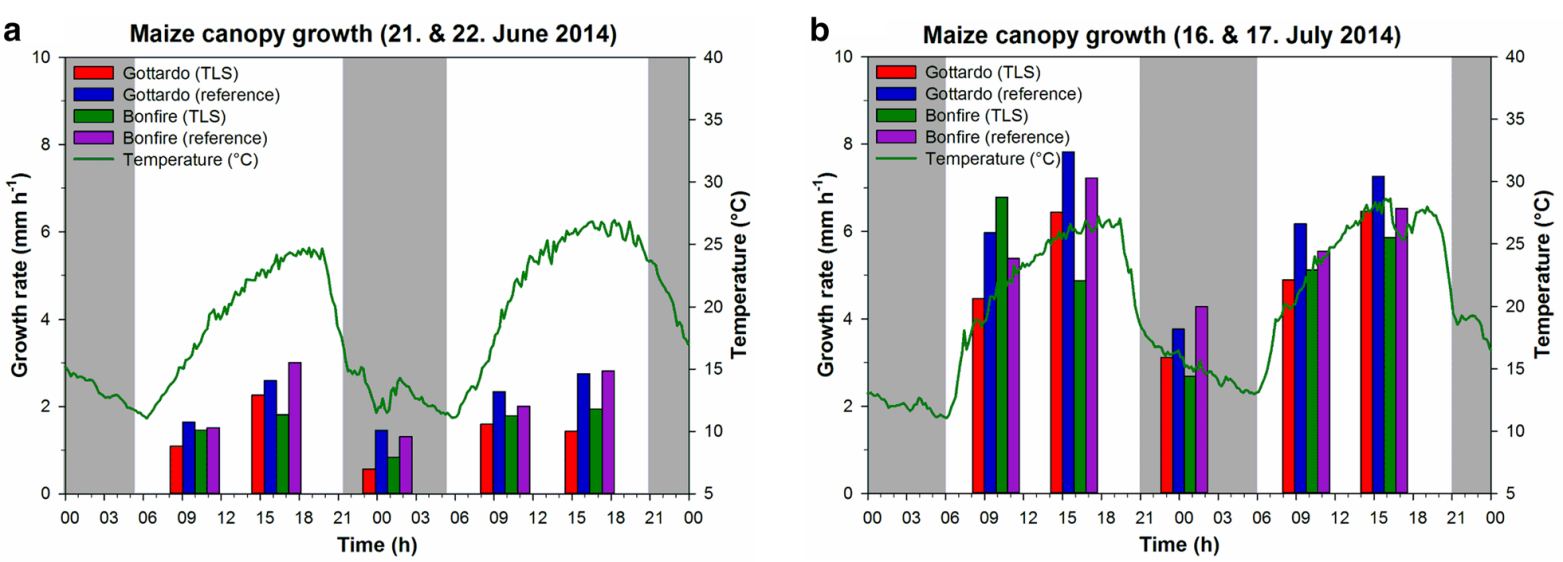
Maize canopy height growth. Canopy height growth (mm h^−1^) of maize computed from TLS (99th percentile of FA_ALLPOINTS_) and measured manually (reference) for the two varieties ‘Gottardo’´ and ‘Bonfire’. Measurements were conducted in the year 2014: **a** 21 and 22 June; **b** 16 and 17 July. Air temperature is shown as a *green line*. *Shaded areas* indicate the period between sunset and sunrise

Maize displayed a diel (24 h) growth pattern that followed temperature (Fig. 6). This growth pattern obtained from TLS was confirmed by manual height reference measurements and was observed during both measurement campaigns in June and July. Highest growth rates were found in the afternoon when the temperature reached its peak and lowest growth rates were observed during the night when temperature was lowest. In the morning, intermediate values for growth rate and temperature were obtained, respectively. No obvious difference in growth was observed between the two genotypes. In July, the growth rate for both genotypes was nearly twice as high as in June. The different growing stages at the two measurement campaigns and also the faster increase in temperature during the morning in July can probably explain the different growth rates. For most of the measurement points, the reference measurements were higher compared to the TLS-derived values.

**Fig. 7:**
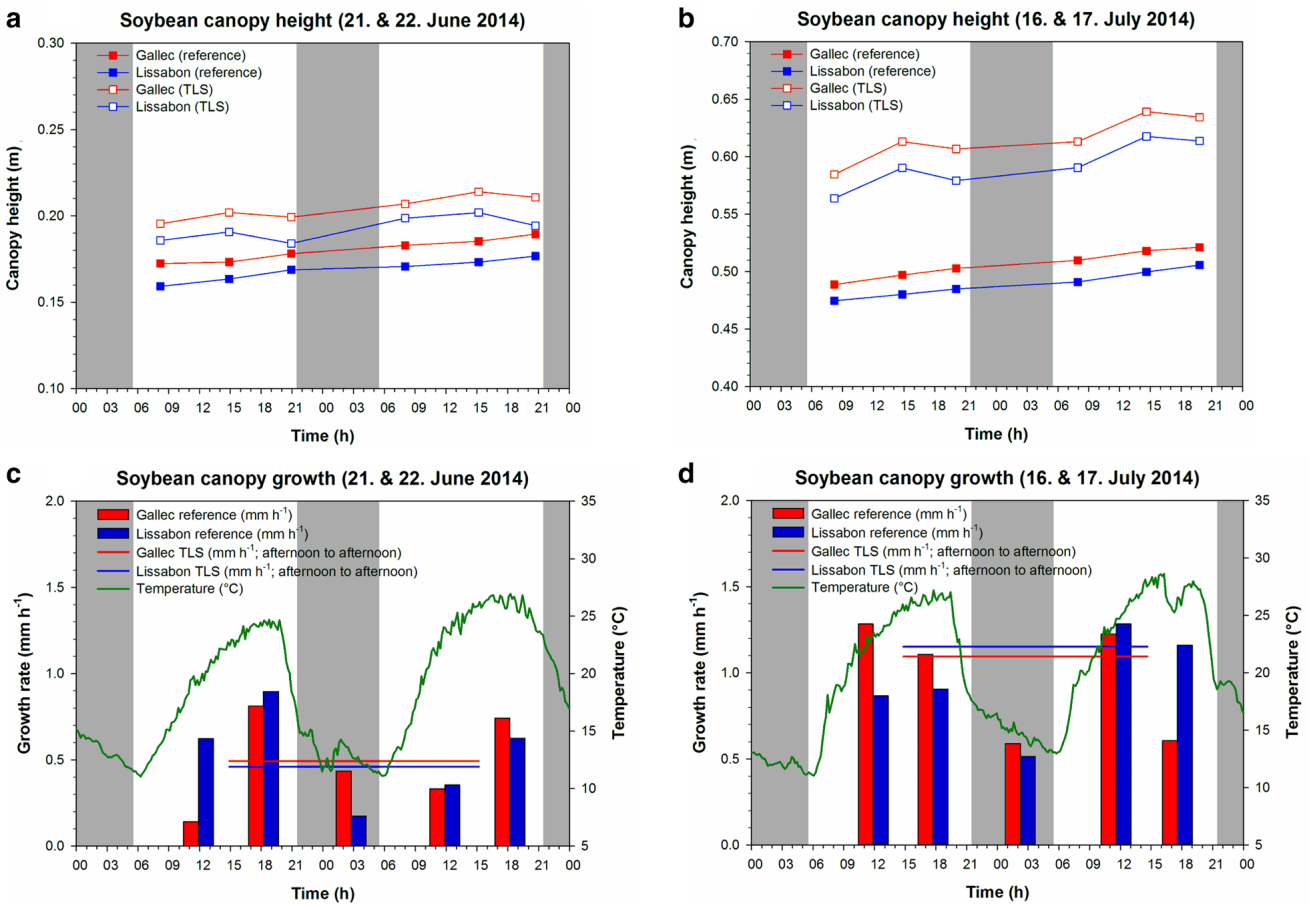
Soybean canopy height and height growth. **a**, **b** canopy height (m) of soybean computed from TLS (99th percentile of FA_ALLPOINTS_) and measured manually (reference) for the two varieties ‘Gallec’ and ‘Lissabon’. **c**, **d** canopy height growth (mm h^−1^) of manually measured soybean plants (n = 10 per genotype and time point) of the varieties ‘Gallec’ and ‘Lissabon’. *Red* and *blue lines* indicated the canopy height growth rate from afternoon to afternoon calculated from the computed TLS data. Measurements were conducted in the year 2014: **a**, **c** 21 and 22 June; **b**, **d** 16 and 17 July. *Shaded areas* indicate the period between sunset and sunrise

**Fig. 8:**
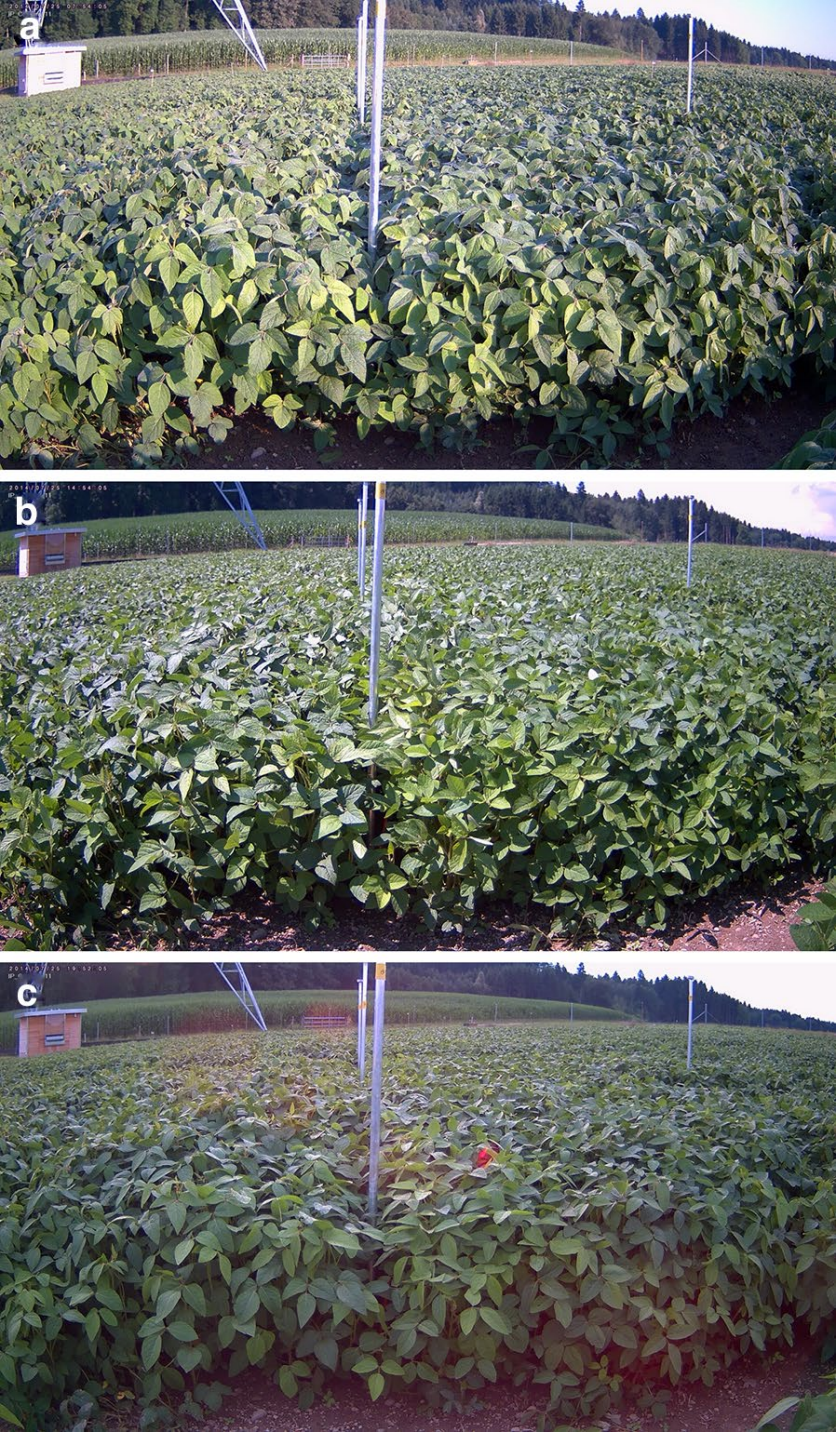
Soybean leaf movement during a day. Images to illustrate movement of soybean leaves and changing canopy height on 25 July 2014: **a** 8 a.m.; **b** 3 p.m.; **c** 8 p.m. The two plots on the *left* and *right* to the closest aluminium rod were sown with the varieties ‘Gallec’ and ‘Lissabon’, respectively

For soybean, the obtained canopy height from TLS increased from the morning to the afternoon but then decreased again towards the evening (Fig. 7a, b). This observed pattern of the canopy height corresponded to the diel (24 h) movement of soybean leaves (Fig. 8) and can be seen very well for the measurement campaign conducted in July. The increase in height from the afternoon of 1 day to the afternoon of the next day clearly indicated canopy height growth. Manual reference measurements showed a continuous increase in canopy height over the measured period of 36 h (Fig. 7a, b). Differences between canopy height values obtained by TLS and manual reference measurements can mainly be explained by different approaches used to obtain these values: P99 of TLS mainly quantifies the height of the uppermost leaf tips; manual measurements quantify shoot height, which is lower. Manual measurements indicated a clear diel fluctuation of height growth with a similar pattern as in maize, largely following the temperature (Fig. 7c, d).

### Plant architectural traits

**Fig. 9:**
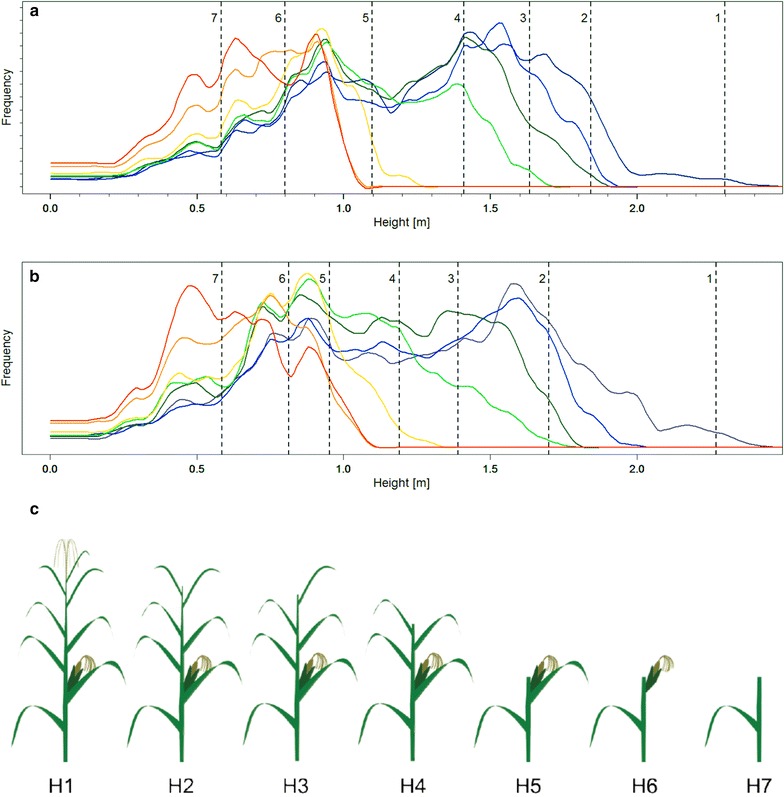
Scan point height distributions (SHDs) and height levels of reference measurements. *Colored lines* show the SHDs for the different height levels derived by step wise cutting of the canopy (*violet* H1, *blue* H2, *dark green* H3, *bright green* H4, *yellow* H5, *orange* H6, *red* H7). *Dashed lines* with numbers stand for the average reference measurements of different plant parts. (*1* height of the whole plant, *2* flag-leaf, *3* second leaf, *4* third leaf, *5* forth leaf, *6* ear-leaf, *7* ear). *Data* are shown for the maize genotypes ‘Bonfire’ (**a**) and ‘Poya’ (**b**). Overview of the different height levels derived by stepwise cutting of the canopy (**c**) (maize drawings adapted from http://www.openclipart.org); for details see Table 2

TLS of the “height level experiment” in maize showed that scanning of the whole, intact canopy revealed positions of the different plant organs in a relatively reliable manner (Fig. 9, violet line). Shoulders (local maxima) in the scan point height distribution (SHD) of the intact canopy corresponded well to leaf and ear positions in the subsequent scans of the “height level experiment” (dashed lines in Fig. 9). Of course, leaves positioned lower in the canopy became more pronounced, when leaves on the top were cut and dismissed. This was true for both genotypes which reflected only small differences between each other.

### Deviations of transformed positions of spherical targets

Small deviations of transformed positions of spherical targets in wheat (0.0084 ± 0.0039 m), maize (0.0042 ± 0.0031 m) and soybean (0.0021 ± 0.0006 m) were achieved (Table 1). These deviations include technical measurement limitations of the laser scanner and potential movement of the positions of spherical targets throughout the field season.

**Table 1:** Overview of the deviations of transformed positions of spherical targets throughout the measurement period

Species	Average deviations of transformed positions (in m ± standard deviation)	Number of transformed sphere positions
*Triticum aestivum*	0.0084 ± 0.0039	24
*Zea mays*	0.0042 ± 0.0031	60
*Glycine max*	0.0021 ± 0.0006	60

## Discussion

So far, most TLS studies conducted very detailed [43] and mostly indoor [39, 44] measurements or they were carried out on large areas in the field [45] with often low spatial resolution. Time intervals between measurements were often in the range of weeks if ever several scans were made and until now, to our knowledge, no one examined the temporal resolution limits of TLS on canopy height growth of crops in the field. With our approach, we fill the gap between these two extremes. We obtained a better resolution as for example in [45] and therefore increase the applicability of TLS for breeding-related phenotyping and precision agriculture. For breeding, many different genotypes planted often on relatively small plots of a few square meters need to be characterized with respect to their performance and their reaction towards alterations of environmental parameters [2, 46]. Further, our approach can be used as ground truth calibration method for new measurement systems from e.g. UAVs developed for precision agriculture [10, 11]. [11] obtained an R^2^ of only 0.7 and a constant underestimation of 0.19 m of the plant height from the UAV-based data compared to manually measured plant heights. In [12] the height measurements from UAV even indoors had a measurement error of above 3.5 cm. UAVs produce wind (downwash) by themselves that can move plant canopies when flying at low altitudes. Therefore, a certain distance of UAVs from the plant canopy is needed to exclude any influence on the plant canopy. Increasing the flight altitude, however, decreases the resolution of measurements and thus also the accuracy of plant canopy reconstructions. Therefore, our high precision method (R^2^ of 0.92 for FA_ALLPOINTS_ for the last date and R^2^ of 0.99 for FA_ALLPOINTS_ using the three dates combined) could be used to calibrate such systems. The fixed position of the spherical targets during the whole season on aluminium rods and solid ground screws is new in plant science under field conditions and at the same time a simple approach, which leads to a high precision of the measurement and therefore allows for comparison of canopy parameters (e.g. canopy height growth) at subsequent time points. In our approach, the spherical targets define a coordinate system that is fixed during the complete season and that can be used to transform scan point clouds from different measurement dates into one and the same reference coordinate system. The small deviations of transformed positions of spherical targets are a strong evidence for the high accuracy of our measurement setup (Table 1). These deviations include technical measurement limitations of the laser scanner and potential movement of the positions of spherical targets throughout the season and are at the same time giving a value for the best achievable accuracy of our TLS approach. In our approach only the spherical coordinates have to be known and no additional expensive device such as a tachymeter or a GPS, as e.g. used by [11], is needed to measure the exact position of targets at each measurement date. Such measurement devices have their inherent technical resolution limits and sources of error that may result in an accumulation of inaccuracies during the season.

By evaluating different FAs for calculating canopy height from TLS data, we could show that in the FA_ALLPOINTS_, the 99th percentile is best suited for computing the wheat canopy height (Fig. 4). This FA has, compared to other studies [29, 45], the advantage that outliers are excluded from calculations and thus the risk of over- or underestimation of the canopy height can be reduced. Another benefit of this FA, compared with FA_MEDIANP99_ is that no potentially important points are a priori excluded from the calculation. The temporal dynamic of the canopy is also clearly visible in the progress of the value of R^2^: a lower canopy is more “susceptible” for underestimation of the canopy height depending on the chosen percentile.

In a recent study, Hammerle et al. [45] investigated the effect of reduced point density of TLS crop surface models of wheat and rye. They examined the effect of a stepwise point reduction on the calculated canopy height from the maximum points or from the 90th percentile (P90) compared with the original resolution and a low resolution scan. However, their low resolution scans and simulated reduced point clouds had only 30–50 points per m^2^. For our purposes, this would have been by far a too low resolution to detect genotypic differences. Moreover, we could show for wheat (Fig. 4) that—in younger growth stages—the real canopy height will be underestimated by using P90 and that using absolute maximum points for canopy height calculation involves the risk of including outliers.

The temporal resolution that can be achieved with our approach depends on the scanned crop. For maize that only shows slight leaf movements, a temporal resolution of several hours can be achieved by scanning e.g. three times per day. In contrast, soybean exhibits a strong diel leaf movement, resulting in a strong change of canopy height during a day (Fig. 8). Thus, soybean is ideally scanned only once per day, after leaves have reached their most horizontal orientation. By taking the difference of the canopy height between 2 days at this time point, the real increase in canopy height will be detected. Otherwise, detected changes in canopy height are a jumble of daily leaf movements and real canopy height growth. Maize and soybean illustrate how important a sound knowledge about physiological processes of a scanned crop is. Growth of maize (Fig. 6) and soybean (Fig. 7c, d) follows temperature. For maize this is no surprise, as it is known from literature, that growth of monocot species follows temperature (e.g. [47]). For soybean, the observed growth pattern with the highest growth in the afternoon is in contrast to the notion that dicot species show their maximal growth activity in the beginning of the day (type 1) or at the end of the day (type 2) [1]. For soybean it was shown in several studies (e.g. [48, 49]) that maximal leaf growth occurs towards the end of the night.

For maize, we could show that plant architectural traits are detectable by TLS with our method (Fig. 9). The obtained scan point height distribution histograms indicate the height position of plant organs, such as leaves and ears and genotypic differences in light penetration properties as potentially affected by number of leaves, leaf area index or leaf angles. Neither for maize (Fig. 6) nor for soybean (Fig. 7) different growth patterns could be detected by TLS for different genotypes in this study. For the precise distinction of genotypes by TLS beyond canopy height detection further studies including more genotypic variance and more measurement points during the season will be needed.

With our TLS approach of data acquisition and data analysis, we established—compared to other TLS studies—a quite simple way of handling TLS data of field crops. Our TLS approach therefore can be considered as a valuable tool to measure the canopy height growth of different crops under field conditions. The high correlation between manually measured and TLS-derived canopy height of wheat is showing the high accuracy of our method (Fig. 4). The fact, that we could measure the diurnal pattern of canopy height growth in maize is another strong evidence for the accuracy of our TLS method (Fig. 6). However, there are some restrictions to perform meaningful measurements. No measurement can be conducted if it rains due to the laser scanner that is not completely weatherproof and also due to technical issues regarding the scattering of the laser beam on raindrops. However, many devices used for field phenotyping cannot be used during rain. During the scanning process, it should ideally not be windy to prevent a blurred point cloud of the scanned crop. The dependence on windless conditions, however, depends on the scanned crop and also on the developmental stage of the crop and the research question. The scanning of crops that are small, stiff and have less surface exposed to wind is less dependent on wind conditions. The scanning of younger and thus normally smaller plants is also less affected by wind. Wind speeds of 2 m s^−1^ are feasible as our approach uses the statistical percentile method and therefore has a certain robustness against deviations caused by wind.

## Conclusion

The TLS approach presented here allows for measuring canopy height growth and architecture of different crops under field conditions with a high temporal resolution, depending on crop species. The approach will therefore be a valuable component of plant breeding programs. It can also facilitate the elucidation of stress-related plant responses in the field in a variety of plants. Furthermore, additional and new plant/crop parameters as for example canopy volume, leaf angle distribution (in the absence of wind) and height positions of key organs such as leaves and ears could be computed and analyzed by accordingly adjusting the scanning resolution and the distances between scanning positions.

## Methods

### Laser scanner

Measurements were performed with a “Faro Focus 3D S 120” laser scanner (Faro Technologies Inc., Laker Mary, USA) (Fig. 1b). The scanner allows the acquisition of point clouds of 7.1 up to 710.7 million points (MP). The number of points corresponds to the resolution of the measurement. Different quality options that differ in the ranging noise and scan rate (Hz) at a certain resolution are available. Scans with higher quality acquire range data with increased observation time and less noise. The scanning range of the device is up to 120 m and the accuracy in 10 m distance is 2 mm. The device uses a laser beam at 905 nm and the “phase shift measurement technology” to detect distances. In this system, infrared laser light is sent out and reflected back to the system. The distance of an object to the scanner is measured by analysing the shift in the phase of the returning beam [50]. The scanner can measure 360° on the vertical axis by rotation of the head of the scanner and 300° on the horizontal axis by a rotating mirror. The scanner was mounted upside down on an elevator tripod (elevator tripod aluminium 3.8 m, 50 kg max. load, VARYTEC, Germany) at a height of about 3.5 m (Fig. 1a). This resulted in typical distances between scanner and canopy of 2–10 m. The point distance of the used resolutions ranged from 0.6 to 1.2 mm at 2 m distance and from 3.1 to 6.1 mm at 10 m distance, respectively (see Table 2 for more details). It is intended to use the scanner on an automated mobile platform [51].

**Table 2:** Overview of the scanned species, dates, measurements per date, scan parameters and reference measurements

Species	Date	TLS measurements per date	Scan resolution/quality	Point distance at 2 m (in mm)	Point distance at 10 m (in mm)	Reference measurement
*Zea mays*	23 Sep. 2013	7	0.5/3x	0.614	3.068	Plant height
*Triticum aestivum*	27 Mar. 2014	1	0.25/3x	1.227	6.136	Canopy height
*T. aestivum*	02 Apr. 2014	1	0.25/3x	1.227	6.136	Canopy height
*T. aestivum*	09 Apr. 2014	1	0.25/3x	1.227	6.136	Canopy height
*T. aestivum*	15 Apr. 2014	1	0.25/3x	1.227	6.136	Canopy height
*T. aestivum*	09 May 2014	1	0.25/3x	1.227	6.136	Canopy height
*Glycine max*	21 Jun. 2014	3	0.5/3x	0.614	3.068	Plant height
*G. max*	22 Jun. 2014	3	0.5/3x	0.614	3.068	Plant height
*G. max*	16 Jul. 2014	3	0.5/3x	0.614	3.068	Plant height
*G. max*	17 Jul. 2014	3	0.5/3x	0.614	3.068	Plant height
*Z. mays*	21 Jun. 2014	3	0.5/3x	0.614	3.068	Plant height
*Z. mays*	22 Jun. 2014	3	0.5/3x	0.614	3.068	Plant height
*Z. mays*	16 Jun. 2014	3	0.5/3x	0.614	3.068	Plant height
*Z. mays*	17 Jul. 2014	3	0.5/3x	0.614	3.068	Plant height

### Setup in the field and data acquisition

Measurements were conducted in the field of the research station for plant science of ETH Zurich in Eschikon, Lindau in 2013 and 2014 (Table 2). Maize (*Zea mays*) and wheat (*Triticium aestivum*) as monocot species as well as soybean (*Glycine max*) as a dicot species were scanned periodically with the laser scanner throughout the season.

Fields were scanned from different positions at the same point in time in regular intervals ranging from several scans per day to weekly scans. White spherical targets (For maize and soybean: 14.5 cm in diameter, Laserscanning Europe GmbH, 39,120 Magdeburg, Germany; For wheat: 30 cm in diameter; do-it-yourself product) were distributed in the scanned area to allow for the later merging of the single scans from the same point in time but from different positions of a field to a scan point cloud. These targets were mounted on aluminium rods (Fig. 1c; 1.52 m in length for soybean and wheat; 3.02 m in length for maize; 3 cm in diameter for all crops) that in turn were fixed to ground screws (80 cm in length, Krinner GmbH, 3272 Walperswil, Switzerland). As spherical targets and aluminium rods are sensitive to environmental influences, they were only positioned in the field during measurement times. The ground screws were positioned within the rows to avoid any contact to machines. Thus, the position of the spherical targets remained constant during the season and defined a fixed coordinate system for all measurements. By transforming scan point clouds to this fixed coordinate system, scan point clouds throughout the season could be aligned for each crop, facilitating the comparison of the canopy at the different measurement points.

In 2013, the scanned maize field had a size of 24 m by 17 m and consisted of 8 plots with a length of 8 m and a width between 5.25–6.75 m corresponding to 8 and 10 rows, respectively. The two varieties ‘Bonfire’ and ‘Poya’ (DSP, Delley Switzerland) were sown each on four of these plots. Eight white spherical targets were distributed over the maize field. A “height level experiment” was performed to test the hypothesis that the scanning of the whole canopy allows for the detection of leaf and ear height levels from maize plants. This was done by scanning a subplot containing four rows of ‘Bonfire’ and ‘Poya’, respectively from four positions on the 23 September 2013. After this, the plants were cut down step wise (removing first the tassel, then the flag leaf, then the second leaf from the top), each cut followed by the next scans (Table 3). With this procedure seven height levels were scanned in total (Fig. 9). Manual height reference measurements were taken on ten maize plants for each variety and height level, respectively.

**Table 3:** Overview of the cut parts and cutting points in the “height level experiment”

Height level	Cut parts	Cutting point
H1	Non	Non
H2	Tassel and flag leaf	Above second leaf
H3	Second leaf	Above third leaf
H4	Third leaf	Above forth leaf
H5	1–3 leaves	Above the ear
H6	Ear leaf	Above the ear
H7	Ear	Below the ear

In 2014, the scanned part of the maize field had a size of 6 m by 6 m and consisted of eight rows with a row spacing of 0.75 m. The two varieties ‘Bonfire’ and ‘Gottardo’ (KWS Saat SE, Einbeck, Germany) were sown each in four rows (Fig. 2b). Five white spherical targets were distributed over the scanned area and at each date, scans from the four corners were carried out at around 6 a.m., 1 p.m. and 7 p.m. (6 p.m. in July). The scanned part of the soybean field had a size of 6 m by 6 m and consisted of four plots with a size of 1.5 m by 6 m. The two varieties ‘Gallec’ (DSP, Delley Switzerland) and ‘Lissabon’ (fenaco Genossenschaft, Bern, Switzerland) were sown each on two plots (Fig. 2c). Five white spherical targets were distributed over the scanned area and at each date, scans from the four corners were conducted at around 8 am, 3 p.m. and 9 p.m. (8 p.m. in July). The wheat field had a size of around 30 m by 40 m (Fig. 2a). Seven white spherical targets were distributed over the whole wheat field and the field was scanned from 16 positions distributed homogeneously over the field. For later analysis only a part of the field (around 24 m by 24 m), including 192 plots with each a size of 1.5 m by 1.7 m, was used. 156 different genotypes were sown in these 192 plots (see [52] for more details).

Manual height reference measurements in soybean and maize in 2014 were taken during the first and the last scan on ten plants per genotype. In maize, the distance from a nail head in the soil next to the plant and the tip of the youngest leaf, which was manually straightened into an upright position, was measured. In soybean the distance from a nail head in the soil next to the plant and the tip of the shoot axis was measured. Manual height reference measurements per plot in wheat in 2014 were taken by holding a yardstick in the canopy at three positions and reading the value.

### Data processing and data analysis

At the beginning of the season a measurement of soil level is done, afterwards measurements for plant heights can be performed. After the automatic detection of the spherical targets, single scans from each measuring date were registered according to the targets and with the use of the inclinometer in the software “FARO SCENE” (Faro Technologies Inc., Laker Mary, USA). Computed scan point clouds were exported as xyz-files (ascii format) and later processed with custom MATLAB^®^ (The Mathworks, Natick, MA, USA) functions. Evaluation was done with an off-the-shelf computer (Intel^®^ Core™ i7-3770 processor, 24 GB installed memory). The software together with a manual and example data can be downloaded from SourceForge (http://sourceforge.net/projects/cahst4tls). To reduce the file size and speed up the subsequent data analysis xyz-files were converted to mat-files (MATLAB^®^, binary data format). In the following the points contained in the 3D point clouds are always called scan points. These are used to calculate height maps whose elements are named pixels. For the generation of height maps percentiles of the z-coordinate were used as a statistically robust method [12, 40]. The stepwise processing and analysis of the point clouds were done as follows:

1. Point cloud transformation to the fixed coordinate system: Sphere coordinates of all scans were manually exported to txt files. They were used to estimate the rigid coordinate transformation for all point clouds to fixed coordinates [53]. The deviations of transformed sphere coordinates of each sphere from different scans were saved to txt-files as they give a value for the best achievable accuracy. The scan point clouds were then transformed to the fixed coordinate system.
  - (in case of soil level measurement) Generation of soil height as a “height image” (H_S_) with 5 mm pixel size (Fig. 3). Therefore the height minimum was first determined on the pixel grid. Gaps were interpolated. The result was median-filtered with a patch size of 21 cm.
  - (in case of plant height measurement) Subtraction of soil level from each point of the point cloud using the appropriate entry of H_S_. The appropriate pixels of H_S_ were found by projection of the point cloud along the z-axis. All points which are projected on the same pixel belong to a column with quadratic base area of 5 mm × 5 mm. They were processed together in the further evaluation by calculating their percentiles (Fig. 3).
2. Selection of regions of interest (ROIs) as individual areas or as a grid (Fig. 2). Border rows were not within the selected ROIs to exclude border effects.
3. Height analyses of the point cloud were carried out with two classes of analysis approaches consisting of three filtering approaches (FAs) in total. The first class consisted of one FA (FA_ALLPOINTS_) and the second class of two FAs (FA_MEDIANMAX_ and FA_MEDIANP99_), respectively:
  - Percentiles of all points (FA_ALLPOINTS_)
    i. Calculation of the percentiles for each ROI by taking every point of the whole scan point cloud within the ROI into account.
  - Patchwise
    i. Taking the maxima [or the xth percentile (Px)] for each 5 mm × 5 mm pixel, a height map H_P_ of the point cloud was calculated (Fig. 3). Points higher than 10 cm were regarded as “plant points”, lower points were neglected.
    ii. Patchwise calculation of percentiles of H_P_ (edge length 15 cm in direction of the row and full row width for maize and 10 cm × 10 cm for soybean and wheat). Number of plant pixels N per patch was saved for later “weighting”.
    iii. Calculation of the weighted median of percentiles per ROI. To calculate the weighted median of a percentile of a ROI each Px of all patches of the ROI was put N times to a list of which the median was calculated. Therefore patches with high plant coverage were stronger weighted than those with low plant coverage. In this study we applied the maximum value and P99 filter (FA_MEDIANMAX_ and FA_MEDIANP99_).
4. Calculation of canopy height and growth or other parameters
